# Role of polar nanoregions with weak random fields in Pb-based perovskite ferroelectrics

**DOI:** 10.1038/srep44448

**Published:** 2017-03-16

**Authors:** M. A. Helal, M. Aftabuzzaman, S. Tsukada, S. Kojima

**Affiliations:** 1 Graduate School of Pure and Applied Sciences, University of Tsukuba, Tsukuba, Ibaraki, 305-8573, Japan; 2 Department of Physics, Begum Rokeya University, Rangpur, Rangpur, 5400, Bangladesh; 3 Department of Physics, Pabna University of Science and Technology, Pabna, 6600, Bangladesh; 4 Faculty of Education, Shimane University, Matsue, Shimane, 690-8504, Japan

## Abstract

In relaxor ferroelectrics, the role of randomly orientated polar nanoregions (PNRs) with weak random fields (RFs) is one of the most puzzling issues of materials science. The relaxation time of polarization fluctuations of PNRs, which manifests themselves as a central peak (CP) in inelastic light scattering, is the important physical quantity to understand the dynamics of PNRs. Here, the angular and temperature dependences of depolarized and polarized CPs in 0.44Pb(Mg_1/3_Nb_2/3_)O_3_-0.56PbTiO_3_ single crystals with weak RFs have been studied by Raman and Brillouin scattering, respectively. The CPs observed in Raman scattering show the very clear angular dependence which is consistent with the local tetragonal symmetry. It is different from the well-known local rhombohedral symmetry with strong RFs for Pb(Mg_1/3_Nb_2/3_)O_3_. In Brillouin scattering, depolarized and polarized CPs show two relaxation processes corresponding to transverse and longitudinal fluctuations of PNRs. The remarkable slowing down towards the Curie temperature was observed for transverse fluctuations in local tetragonal symmetry.

Nowadays, the unusual electromechanical properties of lead-oxide perovskite (ABO_3_) relaxors such as Pb(Mg_1/3_Nb_2/3_)O_3_ (PMN) based Pb(Mg_1/3_Nb_2/3_)_1−*x*_Ti_*x*_O_3_ (PMN-*x*PT), Pb(Zn_1/3_Nb_2/3_)O_3_ (PZN) based Pb(Zn_1/3_Nb_2/3_)_1−*x*_Ti_*x*_O_3_ (PZN-*x*PT), have attracted considerable attention by the colossal piezoelectricity, in relation with the morphotropic phase boundary (MPB) in phase diagrams^1^,^2^,^3^,^4^,^5^. Among these materials, relaxor perovskite PMN-*x*PT single crystals are very promising compound as the candidate of ferroelectric material for electromechanical transducers due to their excellent piezoelectric coefficients, ultrahigh strain levels with low hysteresis, high dielectric constant, high electro-optic coefficients, and high electromechanical coupling factor^6^.

The compositional disorder, i.e., the disorder in the arrangement of different ions at a crystallographic equivalent site, is a common structural property of relaxor ferroelectrics. It is reported that the differences of two B-site cations on the same kind of lattice sites is the source of quenched random fields (RFs) and play a key role in establishing the relaxor state^7^,^8^. The appearance of polar distortion is a consequence of the ion off-centering that is a common property of the most of perovskite ferroelectrics. Unlike KTa_1−*x*_Nb_*x*_O_3_ (KTN), where Nb is only the off-centered positive ion, PMN-*x*PT represents a more difficult case. It was reported that in PMN-*x*PT, not only the ions of B-site, Mg, Nb, and Ti are shifted by ~0.1–0.2 Å, but also Pb, the ion of A-site, is off-centered by ~0.387–0.398 Å^9^. The displacement of Pb ions take places from their high symmetry site along [100] direction and that of B-site ions tend to shift along [111] direction^9^,^10^. At high temperatures, all the off-centered ions are expected to switch relatively freely between their positions and the time average structure remains cubic^11^. At a slightly lower temperature, their positions and motions become correlated, giving rise to the formation of polar nanoregions (PNRs). It is well-accepted that the relaxor ferroelectrics show three characteristic temperatures: Burns temperature *T*_B_, when the lifetime of polar fluctuations becomes longer than the inverse optical phonon frequency; intermediate temperature T*, when permanent PNRs appear; and cubic-tetragonal phase transition temperature *T*_C-T_.

Although a lot of studies have been performed on PMN-*x*PT systems, till now it remains to be confirmed whether the maximum limit of PT content up to which the PNRs persist. Z. -G. Ye *et al*. showed that the substitution of Ti^4+^ ions for Mg^2+^/Nb^5+^ ions can decrease the effect of RFs in PMN-*x*PT^12^. Recent neutron diffraction study on PMN-60PT showed very small diffuse scattering, suggesting the absence of static PNRs^13^. However, the diffraction method cannot detect dynamic PNRs. In addition, some phenomena typically related to the relaxation of dynamic PNRs, namely, the intense Brillouin quasielastic scattering, the softening of the longitudinal acoustic mode, and the deviation from the Curie-Weiss law above the Curie point were observed in PMN-55PT^14^,^15^,^16^. Very recent Raman scattering study on a PMN-56PT single crystal also revealed the existence of PNRs^17^. In this situation, another approach may be to investigate the dynamics of PNRs with weak RFs and corresponding relaxor properties in more detail.

Since the polarization fluctuations of PNRs manifest themselves as a central peak (CP) in a low-frequency inelastic light scattering (ILS) spectrum, therefore, a CP is important to understand the dynamics of PNRs. In this respect, low-frequency ILS can be a very useful tool, because it is very sensitive to local symmetry breaking related to heterogeneity among the few techniques that can be used to directly probe the local symmetry and structure. Therefore, to identify the local structural symmetry of PNRs, the angular dependence of Raman scattering (one of ILS) combined with Raman tensor analysis can be a very powerful technique, because it measures the scattering intensity with respect to the polarization direction of an incident light. Recently, the local symmetry of CPs and some other Raman modes in a paraelectric cubic phase related to rhombohedral PNRs were identified by the angular dependence of Raman scattering in Li-doped KTN^18^ and PMN^19^ single crystals. The angular dependences of Raman spectra of PT and PMN-33PT were also measured in the tetragonal phase to identify the symmetry of the modes^20^,^21^. Very recently, the phase coexistence in the PMN-*x*PT close to MPB composition has been inferred from the inspection of the angular dependence of Raman scattering^22^. However, as far as we know, the angular dependence of the Raman spectra of PT rich PMN-*x*PT, especially focusing on CPs, in a paraelectric cubic phase is not reported yet.

To observe the variation of the diagonal and off-diagonal elements in the Raman tensor in the vicinity of a phase transition point, it is necessary to measure both the polarized (VV) and depolarized (VH) scattering separately^18^. So far, the low-frequency ILS studies on a phase transition have been performed by using VV or unpolarized light scattering^14^,^23^,^24^,^25^,^26^,^27^,^28^. Above the Curie temperature, very few earlier studies observed the strong VH scattering spectra^29^,^30^,^31^. Y. Nakata *et al*. carried out VV and VH scattering of a phase transition by Brillioun scattering in PZN-11PT single crystals^29^. They observed a strong VH-CP in the vicinity of a phase transition temperature, and ascribed its origin to a *E*-symmetry polarization fluctuation of PNRs. In pure PZN, Y. Gorouya *et al*. observed almost the same intensity in both the VH- and VV-CPs^32^ and they discussed the slowing down mechanism towards the transition point only by the results obtained from the VH-CP.

The dynamic pair distribution function (PDF) of PMN observed by pulsed neutron inelastic scattering indicates that the local [100] shifts are correlated in the medium range and on an average the Pb ions shift along [111] directions, therefore the PNR has rhombohedral *R3m* symmetry^33^. However, the composition dependences of Raman scattering^25^,^26^ and electric field induced polarization^34^ measurements on PMN-*x*PT revealed that the stability of local rhombohedral symmetry is deteriorated by adding PT to PMN. From the characteristic jump of the Raman frequencies and linewidths in the frequency range 700–800 cm^−1^, it was proposed that at high PT contents, the local symmetry of PNRs changes from rhombohedral *3* *m* to tetragonal *4 mm*^25^. In addition, the first principles result showed that at high PT contents, the collinear [100] Pb atom distortions form short Pb-O bonds, maximize dipole alignment, and minimize local Pb-B cation repulsion by avoiding the Ti cations located along the [111] direction^9^. D. La-Orauttapong *et al*. also measured the composition dependences of neutron scattering studies on PZN-*x*PT and suggested that with increasing the PT content, the local polarization direction remains unchanged along [111] direction, while the static PNRs grow preferentially very closer to [100] direction^35^. In PZN and KTN, on cooling, the B-site cations was shown to restrict progressively in fewer [111] directions which results in the local tetragonal symmetry^27^,^30^. However, in spite of extensive studies on the dynamical aspects of PNRs, the exact knowledge of static and dynamic features of PNRs with weak RFs and their local symmetry is still an intriguing topic. Therefore, the detailed polarization analysis on the VV- and VH-CPs in a cubic phase, which is directly related to PNRs, is important and also necessary in Pb-based perovskites with weak RFs.

In the present study, the physical origin of the CPs in two different scattering geometries has been discussed by the angular dependence of Raman scattering in a paraelectric cubic phase. In addition, the temperature dependence of Brillouin scattering has been performed to discuss the physical origin of the intense VH-CP over the VV-CP in terms of polarization fluctuations of PNRs and their slowing down mechanism.

## Results

### Angular dependence of CP in Raman scattering

**Figure 1:**
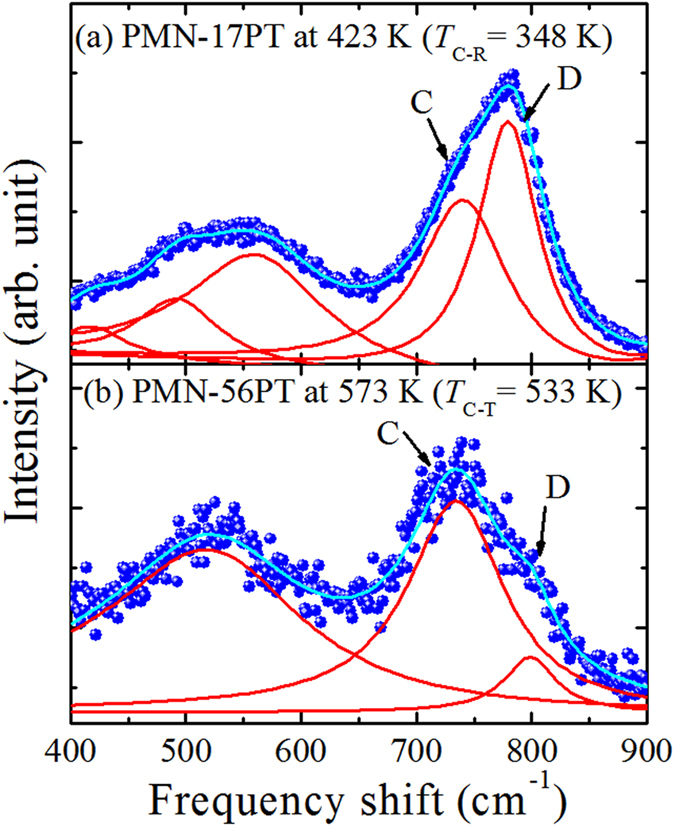
Raman scattering spectra of (**a**) PMN-17PT and (**b**) PMN-56PT single crystals at backscattering geometry along [100] direction in a paraelectric cubic phase in the frequency range between 400–900 cm^−1^.

**Figure 2:**
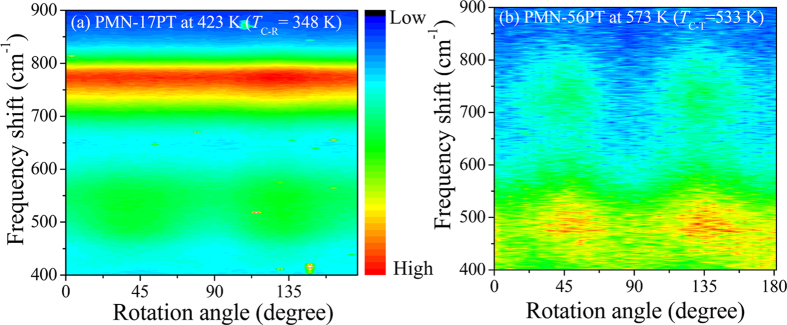
Contour color map of the angular dependences of Raman scattering spectra of (**a**) PMN-17PT and (**b**) PMN-56PT single crystals at VH scattering geometry in a paraelectric cubic phase.

In PMN-17PT, the ferroelectric phase transition from cubic to rhombohedral, *T*_C-R_ was observed at 348 K (data is not shown here), whereas, in PMN-56PT the cubic to tetragonal ferroelectric phase transition, *T*_C-T_ was 533 K (discussed in later). Therefore, to discuss the local symmetry of PNRs and the effect of Ti-concentration on local symmetry of PNRs, we measured the Raman spectrum of PMN-17PT and PMN-56PT in a paraelectric cubic phase as shown in Fig. 1. From Fig. 1, it is clearly seen that in the range between 400–900 cm^−1^, the frequency and linewidths of each Raman mode show significant changes. On the other hand, the angular dependences of the modes within the 400–900 cm^−1^ are shown in Fig. 2. It is remarkable that the modes denoted by C and D do not shows the angular dependences in PMN-17PT while in PMN-56PT these two modes show very clear angular dependences.

These two observations can be owing to the change of local symmetry upon Ti addition. Therefore, by considering the composition dependence of Raman scattering^25^,^26^, first principles^9^, field dependence of polarization and strain measurements^34^, and the present Raman scattering results, it is reasonable to assume that the local symmetry of PNRs is tetragonal with the point group *4 mm*.

**Figure 3:**
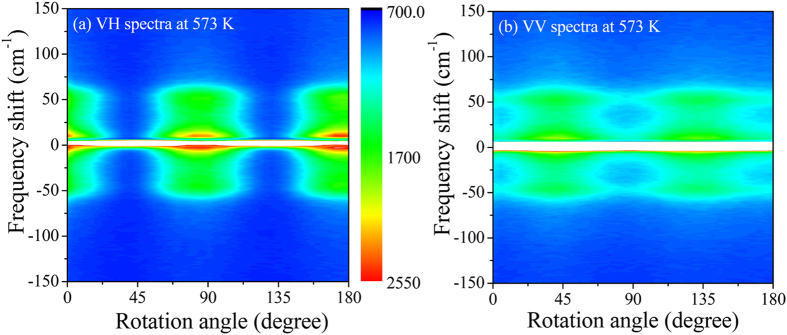
Contour color map of the angular dependences of Raman spectra of PMN-56PT in (**a**) VH and (**b**) VV scattering geometries at 573 K (T_C–T_ = 533 K).

Figures 3(a) and (b) show the contour color map of the Raman scattering intensity of PMN-56PT single crystals as a function of rotation angle in both the *x(yz*)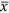 (VH) and *x(zz*)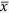 (VV) scattering in a paraelectric cubic phase at 573 K. The prominent CP in both the VH and VV spectra demonstrates the existence of PNRs in PMN-56PT single crystal. In local 4 mm symmetry, we expect that at 0 degree, VV scattering measures *A*_1_(*z*)-symmetry excitations, which is related to the ionic motion parallel to the polar axis, and VH scattering measures *E(x,y*)-symmetry excitations, which is related to the ionic motion on a plane perpendicular to the polar axis.

For quantitative analysis, all the Raman spectra were fitted with the assumption of a Lorentzian CP and the other vibrational peaks by spectral response functions of a damped harmonic oscillator (DHO) modified by the Bose-Einstein factor as follows^36^,


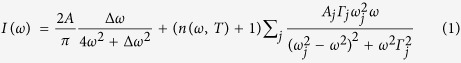


here *A* and Δω are the intensity and width of CP, respectively. *n(ω, T*) is the Bose-Einstein population factor. *A*_*j*_, *Γ*_*j*_, and *ωj* are the intensity factor, damping constant and the mode frequency, respectively, of the *j*th optical phonon mode. The light scattering intensity of phonon modes is proportional to the response of the sum of DHO multiplied by *n(ω, T*)^37^,^38^,^39^. On the other hand, a CP is considered as a relaxation process, therefore, the light scattering intensity of relaxation processes is proportional to the sum of a Debye response multiplied by *n(ω, T*), and is approximated by the sum of a Lorentzian function.

The Raman intensity depends on the Raman tensor and scattering geometry^38^ which can be expressed as:


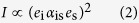


where *e*_i_ and *e*_s_ are the unit polarization vectors of the electric field of the incident and scattered beam, respectively, while *α* represents the Raman scattering tensor of the studied band.

In the tetragonal symmetry, the local polarization direction in PNRs fluctuates along the equivalent [100] directions in cubic coordinates. Therefore, the Raman tensor calculations were performed with tetragonal *4 mm (C*_4v_) symmetry. The observable Raman tensors in *x(yz*)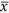 and *x(zz*)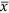 scattering geometries in tetragonal coordinates are presented below. The quantity in parentheses represents the polarization direction of the modes.


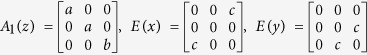


Each Raman tensor components are complex quantities expressed by a = 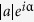. The angular dependence of Raman intensity in cubic coordinates is suggested to be proportional to


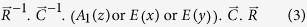


where 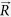 is the coordinate rotation matrix about the cubic [100]_c_ axis,


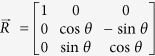


**Figure 4:**
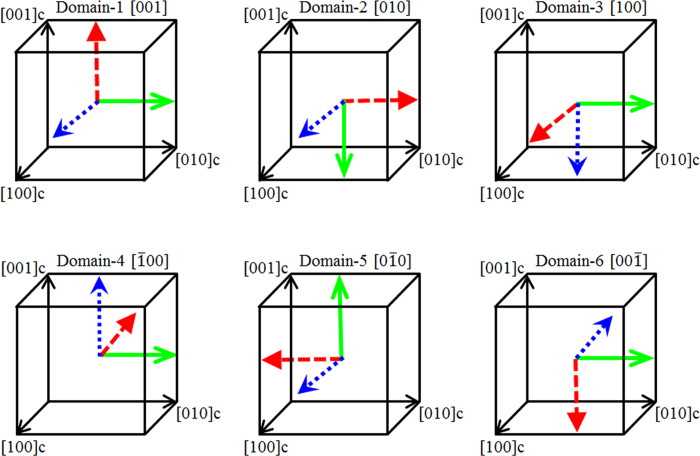
Six equivalent orientations of tetragonal domains with respect to cubic coordinates. Blue, green and red arrows denote the crystallographic *x, y*, and *z* axes, respectively.

The tetragonal symmetry has six equivalent polarization directions as shown in Fig. 4. Here the local tetragonal regions are considered to be randomly oriented.

The angular dependences of Raman spectra in cubic coordinates were calculated in multidomain state, where the contributions of all domains were summed up equally. Since the tetragonal coordinates differ from the experimental ones used for polarization rotation, the Raman tensor for *4 mm* symmetry in tetragonal coordinates must be modified according to the experimental coordinates, which are equivalent to cubic coordinates. In Eq. (3), matrix 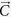 is the transformation matrix corresponding to Raman tensor modification from tetragonal to cubic coordinates. The matrix 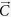 for the six domain directions along [100]_C_ of the local tetragonal region are given below:


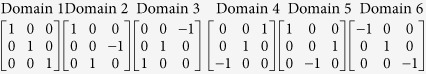


After transforming the Raman tensor elements from tetragonal to cubic coordinate systems by using Eq. (3), we calculated the Raman scattering intensity for the *E*-modes of PNRs. The intensity of the *E(x*) and *E(y*) modes was calculated independently for all the six domains and then summed up (*I*_*E(x,y*)_ = *I*_*E(x*)_ + *I*_*E(y*)_), because these two modes appear at the same frequency of the Raman spectra. Thus, one can obtain the values of the intensity of the *zz* (VV) and *yz* (VH) components for the *E(x,y*) mode as


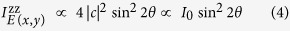



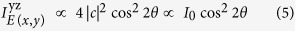


Similarly, for the *A*_1_(z) mode as


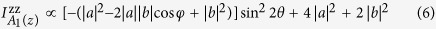



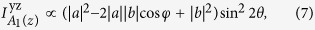


where 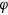 = arg(*b*) - arg(*a*) represents a relative phase difference between two Raman tensor components of *A*_1_(*z*). The above equations are suitable to fit the rotation angle dependence of Raman scattering intensity. Therefore, it is reasonable to identify the symmetry of a mode by Eqs (4, 5, 6, 7).

**Figure 5:**
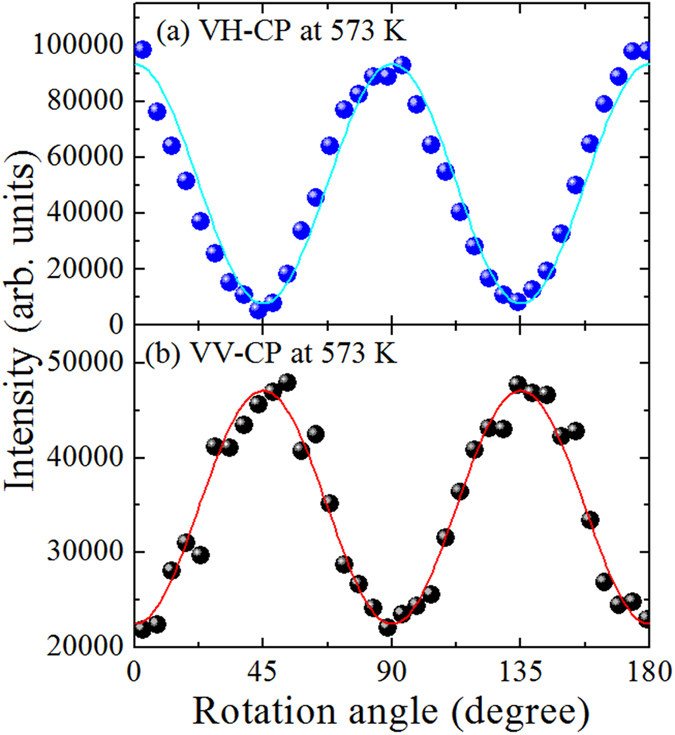
Angular dependences of CP intensity in (**a**) VH and (**b**) VV scattering geometries, where closed circles and solid lines represent the observed and calculated intensities, respectively.

The angular dependences of the VH- and VV-CPs intensities measured at 573 K, with a step interval 5 degree, are shown by the closed circles in Figs 5(a) and (b), respectively. It is clearly seen that the Raman intensities of VH- and VV-CPs strongly depend on the rotation angles. The solid lines denote the fitting results using Eqs (5) and (4), respectively.

### Temperature dependence of CP in Brillouin scattering

**Figure 6:**
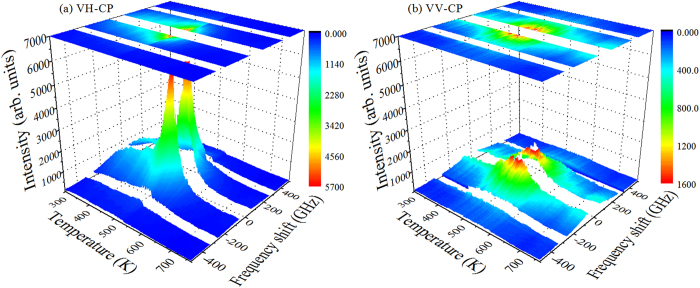
Contour color maps of the (**a**) VH- and (**b**) VV-CPs of PMN-56PT single crystals vs. temperature and frequency in Brillouin scattering spectra at θ = 0°.

In order to observe the quasi-elastic CP, Brillouin scattering is a very convenient probe. The presence of a CP in the Brillouin spectrum can reveal the characteristic relaxational polarization fluctuations associated with the reorientation of PNRs. In this respect, the temperature dependences of the VH- and VV-CPs were measured to investigate the relaxation process of PNRs. Figures 6(a) and (b) show the contour color maps of the VH- and VV-CPs intensity versus temperature and frequency shift in PMN-56PT single crystals.

**Figure 7:**
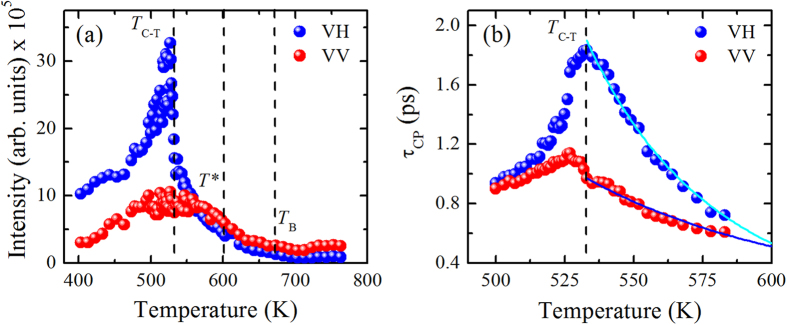
The temperature dependences of (**a**) the CPs intensity and (**b**) relaxation time of PMN-56PT in Brillouin scattering measured at VH and VV scattering geometries. The solid lines represent the best fit by Eq. (9). The values of the activation energy (*H*_0_) and the attempt frequencies *υ*_0_ ( = 1/2π*τ*_0_) determined from the fittings are *H*_0T_ = 0.52 eV and *υ*_0T_ = 8.8 × 10^11^ Hz for CP in VH scattering, and *H*_0L_ = 0.26 eV and *υ*_0L_ = 5.5 × 10^11^ Hz for CP in VV scattering, respectively.

It is seen that the intensity of the CPs increases with decreasing temperature down to 533 K. In addition, above *T*_B_, the important feature is the strong and narrow scattering in the VV geometry accompanied by relatively very weak scattering in the VH geometry. The intensity of the VH- and VV-CPs which were obtained by fitting with the Voigt function, are displayed in Fig. 7(a). Upon cooling, CPs show drastic changes at three characteristic temperatures: (i) the VH-CP begins to increases at about *T*_B_~673 K due to the appearance of dynamic PNRs, (ii) below T*~603 K, the significant increase of intensity in both components pointing out the local phase transition of PNRs; simultaneous appearance of the diffuse neutron scattering^40^ allows us to associate T* with the nucleation of the permanent PNRs, and (iii) a sudden change of the intensity at *T*_C-T_~533 K indicates a structural phase transition from cubic to tetragonal symmetry. In our present backward scattering geometry, it is seen from Fig. 7(a) that below T*, the intensity of the VH-CP increases rapidly while that of the VV-CP increases gradually. Therefore, the phenomenon observed here for PMN-56PT is rather exceptional and also interesting.

To describe the dynamics of the CPs, the relaxation time was determined by assuming the Debye-relaxation process as


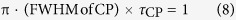


where 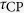 is the relaxation time of the CP. Temperature dependences of *τ*_CP_ for both the VH-and VV-CPs are shown in Fig. 7(b). It is found that the temperature dependence of *τ*_CP_ estimated from the width of the VH-CP is larger than that from the VV-CP.

It was pointed out that the semiclassical tunneling model contains the physically unreliable assumption for relaxor (the potential barrier is invariant with temperature)^28^. Therefore, we considered the modified superparaelectric model^28^, where the size of the microregions and related potentials are assumed to be temperature dependent. According to this model, the activation energy *H* is considered to change as *H*_0_(*T*_B_-*T*)/*T*_B_, where *H*_0_ is the activation energy extrapolated to temperature of absolute zero. Thus, the thermal energy *k*_B_*T* and the relaxation time of the polarization switching *τ*_CP_ are related in the following way:


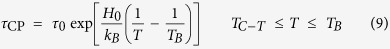


The above formula suggests that the activation energy barrier separating different polarization states of PNRs is proportional to the volume of PNRs which grows upon cooling^28^.

## Discussion

The periodic pattern of the scattered light intensity with the rotation angle observed for the PMN-*x*PT sample is attributed to the change of domain orientations relative to the polarization direction of the incident beam. Since the dependence of Raman intensity on the phase difference originates from a term which is proportional to the product of two different Raman tensor components^41^, therefore, it is seen from Eqs (4)–(7), 16, 17, 18, only the *A*_1_(*z*)-symmetry excitations will have the phase differential contributing to the susceptibility of the polarized angle. From Eq. (7), it is seen that *A*_1_(*z*)-symmetry implies zero Raman scattering intensity for the VH configuration when the incident plane of polarization is parallel (θ = 0 degree) or perpendicular (θ = 90 degree) to the polar axis. The maximum signal should occur at θ = 45 degree. In contrast, we have observed the strongest Raman signal in the VH configuration for θ = 0 degree. Therefore, the observed experimental results can be well reproduced by the Eq. (5), which was obtained by summing the intensity of *E(x*) and *E(y*) modes of PNRs. The solid line in Fig. 5(a) represents the best fitted curve by Eq. (5) which demonstrates a good agreement between the experiment and calculation, suggesting that the VH-CP can be assigned to the tetragonal *E(x,y*)-symmetry of PNR. In a similar way, Taniguchi *et al*. also assigned the local rhombohedral *E(x,y*)-symmetry of PNRs in pure PMN^19^. On the other hand, in VV configuration, at θ = 0 degree and 90 degree, the intensity of the *A*_1_(*z*)-symmetry should be maximum. While, at θ = 45 degree, both *E(x,y*)- and *A*_1_(*z*)-symmetries are active. Since, the intensity of the *E(x,y*)-symmetry is maximum at θ = 45 degree, therefore the angular variations show the *E(x,y*)-symmetry of PNRs. However, we observed a strong background in the VV scattering. The ratio between the background to maximum intensity in the VH scattering is around 8%. And the corresponding ratio in the VV scattering is around 46%. Therefore, the strong background in the VV scattering may stem from the *A*_1_(*z*)-symmetry of PNRs. We have calculated the ratio of the Raman tensor components (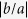 = 1.23) and phase difference (*φ* = 10 degree) from the strong background in the VV scattering by using Eq. (6) for the *A*_1_(*z*)-symmetry of PNRs. According to this result, VH-CP belongs to *E(x,y*)-symmetry and VV-CP belongs to *A*_1_(*z*)- and *E(x,y*)-symmetries while *A*_1_(*z*) is dominant.

In this step, the VH- and VV-CPs in the PMN-56PT single crystals have been discussed by the results obtained from the Brillouin scattering. Both the VH and VV scattering measures the change in the off-diagonal and diagonal contribution of the dielectric tensor and thus suitable for the study of PNR dynamics. Cooling from the high temperature above *T*_B_, as seen from Fig. 7(a), the important feature is the strong and narrow CP in the VV geometry accompanied by relatively weak CP in the VH geometry. This result indicates that, at high temperature range above *T*_B_ where there is no PNR, the off-center ions can reorient freely without restriction. Rapid 180° reorientations of the off-center ions causes the dominance of the VV component of CP over the VH component due to symmetry consideration^40^. This feature is similar to the case of pure PMN and PZN-PT which display a persisting VV component of CP at high temperatures up to 900 K^42^,^43^,^44^. Lowering of the temperature leads to the appearance of the polar clusters near *T*_B_, restrictions of the 180° reorientations, where the intensity of CP begins to increase. The growth of the CP in the VH scattering geometry at about *T*_B_ indicates that off-diagonal components of the polarizability tensor develop around this temperature and therefore, influence the VH scattering. Near T*, the intensity of the CPs increases rapidly indicating the local phase transition from dynamic to static PNRs begins to take place. At this temperature, the permanent PNRs appear with local strain fields. Upon further cooling, both the VH- and VV-CPs increase toward *T*_C-T_ which may be attributed to the increase in size of PNRs as well as to the correlation between PNRs due to the enhanced ferroelectric instability. Although both the VH- and VV-CPs increase as the temperature approaches to *T*_C-T_, the intensity of the VH-CP increases rapidly while that of the VV-CP increases gradually.

**Figure 8:**
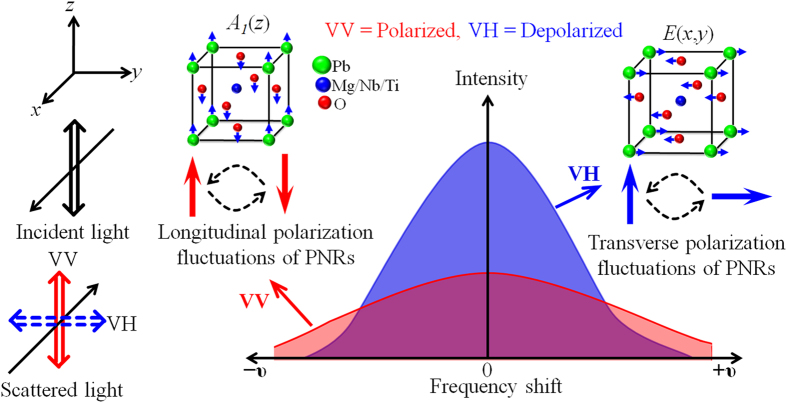
A schematic illustration of the VH- and VV-CPs caused by polarization switching of PNRs under the condition of fixed B-site cations.

It is well known that the VH scattering arises from non-totally symmetric vibrations, and in particular oxygen octahedral rotations^30^. Thus, the rapid change in the VH-CP intensity confirms the contribution of oxygen octahedral rotations to the relaxor dynamics. A similar result was obtained in other relaxor ferroelectrics^30^,^31^ and it was suggested that on cooling, the correlation between off-centered ions in PNRs enhanced and their motion restrict in fewer equivalent [111] orientations, gradually losing the symmetric 180° fluctuations. In PMN-56PT, this mechanism is rather complicated because of the displacement of local Pb ions along [100] direction, and the B-site cations along [111] direction^9^,^10^. It was reported earlier that near T*, the B-site cations move preferentially among the four sites in a plane leading to an effective displacement along [100] direction^27^. Therefore, when the cubic phase will be viewed from any of its fourfold axes, then an average local tetragonal structure will be seen by the correlated B-site cations displacement. In a paraelectric cubic phase, a similar symmetry breaking at the B-sites was observed^45^,^46^. Therefore, now the correlations of the Pb displacements will affect the number of equivalent sites for the B-site cations. Since on cooling, Pb-Pb, and Pb-B correlations become stronger, therefore their motion will gradually restrict in [100], [010] and [001] directions similar to those observed in other relaxors where the motion were restricted in fewer equivalent [111] directions^30^,^31^,^43^. The switching among [100], [010] and [001] polarization directions can enhance the transverse polarization as indeed observed from the strong increase of VH scattering. The intense VH-CP over the VV-CP was also observed in PZN-11PT and attributed to the *E*-symmetry fluctuations of PNRs^29^. However, the authors did not describe the difference between the CPs observed in two different scattering geometries. Figure 8 represents a schematic illustration for the VH- and VV-CPs based on the switching of PNRs.

In lead based relaxor ferroelectric materials, especially in PMN-*x*PT, one of the important questions arises on PNRs about the upper limit of the PT content up to which the PNRs exist. A very weak diffuse scattering was observed due to the absence of PNRs in PMN-60PT single crystals^13^. However, Vakhrushev *et al*. reported on the basis of their high energy resolution neutron spin-echo spectroscopy results that the diffuse scattering in PMN is purely elastic and therefore, can be associated only with the static PNRs, not the dynamic one^47^. It was further suggested that the dynamic PNRs can be observed only in the case of very small scattering vectors which are inaccessible in the neutron and X-ray experiments^47^. Recent dielectric, Raman and Brillouin scattering studies on PMN-*x*PT single crystals with high-PT concentrations showed the relaxorlike behavior, indicating the possibility of the existence of PNRs in this high-PT composition^14^,^15^,^16^,^17^,^25^,^26^. In addition, we have found a quasielastic CP centered near zero frequency in a PMN-56PT single crystal, which can be the direct evidence of dynamic PNRs.

The slowing down of relaxation time in dynamic PNRs is an intriguing topic in relaxor ferroelectric materials. In pure PMN, Vakhrushev *et al*.^48^ and Al-Zein *et al*.^49^ reported that near *T*_B_, a narrow CP appears due to a special transition or a change of dynamics from soft mode to PNRs. Therefore, below *T*_B_, the dynamics is governed by a relaxation of the PNRs^48^. In order to observe the relaxation process inside the PNRs, we measured the relaxation time of CP in both the VH and VV scattering geometries. Interestingly, we observe a very clear difference in relaxation times below *T*_B_ as shown in Fig. 7(b). To explain the difference between the relaxation times observed in VH- and VV-CPs, we consider the flipping motions of the polarization inside a PNR, as suggested by Tsukada *et al*.^50^. The longitudinal fluctuations occurs without any change of local shear strain in the PNRs while the local shear strain changes in transverse fluctuation^51^,^52^. Therefore, the faster relaxation process in VV scattering can correspond to the longitudinal fluctuations of PNRs, while the slower relaxation process in VH scattering can correspond to the transverse fluctuations of PNRs. It is because the motion accompanying shear strain can be slower than that of accompanying longitudinal strain^51^,^52^. As a consequence, the activation energy of the longitudinal fluctuations can be smaller than that of transverse fluctuations.

In order to determine the activation energy of PNRs, we have fitted the relaxation times in both the VH- and VV-CPs by Eq. (9) as shown in Fig. 7(b). The values of the activation energy, *H*_0_, determined from the fittings are *H*_0T_ = 0.52 eV for CP in VH scattering and *H*_0L_ = 0.26 eV for CP in VV scattering. *H*_0T_ and *H*_0L_ correspond to the activation energy of transverse and longitudinal polarizations switching, respectively. It is seen that the activation energy is double in the VH scattering compared to that of VV scattering. The higher value of the activation energy is attributed to the fast growth of PNRs since the activation energy is assumed to be proportional to the volume of PNRs in this model. As a result, transverse polarization switching tends to freeze towards *T*_C-T_ in order to stabilize the tetragonal structure. The values of the observed *H*_0_ are plausible as they are of same order of magnitude as those obtained for PMN-35PT and PZN-20PT^28^,^53^. On the other hand, the values of the attempt frequencies *υ*_0_ ( =  1/2π*τ*_0_) are *υ*_0T_ = 8.8 × 10^11^ Hz and *υ*_0L_ = 5.5 × 10^11^ Hz, corresponding to transverse and longitudinal fluctuations, respectively. Since, the activation energy in transverse fluctuation is higher, therefore, the attempt frequency should be higher compared to longitudinal one as indeed observed from the fittings. These values are in the order of typical Debye frequencies and therefore, justify the fitting procedure based on the modified superparaelectric model.

In summary, we have studied Raman and Brillouin scattering on Pb-based ferroelectrics with weak RFs focusing on CPs. The depolarized (VH) and polarized (VV) CPs observed in Raman spectra indicate the local tetragonal symmetry of PNRs with the point group *4 mm* which is different from local rhombohedral R*3m* symmetry with strong RFs. In Brillouin scattering, at around *T*_B_, the motion of the off-center ions gives rise to the reorientational dynamics of local polarization in PNRs which was seen directly through the evolution of the VH-CP. The switching motion of local polarization in PNRs was gradually restricted towards [100], [010], and [001] directions upon cooling, leading to the strong increase in the VH-CP intensity. By the analysis of the modified super paraelectric model for the VH- and VV-CPs, the higher activation energy of the transverse fluctuation was obtained in the VH-CP. Due to the higher activation energy, the remarkable slowing down of the transverse fluctuation towards *T*_C-T_ was observed. The present study will give a new insight into the role of PNRs in not only Pb-based relaxors but also disordered materials with RFs.

## Methods

### Raman scattering

The perovskite single crystals PMN-56PT and PMN-17PT were grown by modified Bridgman method^54^. Samples were cut into (100)-oriented platelets, which were polished to optical quality. The spectra were measured in back scattering geometry^55^. The sample was put inside the temperature controlled stage (Linkam) on the *xyz* mapping stage (Tokyo Instruments) installed in the microscope (Olympus). Linearly polarized incident light from a diode-pumped solid-state laser (Coherent) with a single-frequency operation at 532 nm and a power of 150 mW traveled to the sample through a polarization rotation device (Sigma Koki) equipped with a broadband half-waveplate (Kogakugiken). Corresponding to the rotation of the half-waveplate by θ/2 with respect to the optical axis, the polarization plane of incidence is rotated by θ degree. Moreover, the polarization direction of scattering light, propagating in opposite direction of incident light, is rotated by - θ degree. The intense elastic scattering is eliminated by two volume Bragg gratings so called “ultra narrow-band notch filters” (Opti Grate). The inelastic scattering light was dispersed by a single-monochromator (Lucir) and the dispersed component was detected by using a charge coupled device (CCD, Andor). In the present study, the polarizer was set to be parallel (VV) and cross nicol (VH) geometries, respectively.

### Brillouin scattering

A micro-Brillouin scattering system with a 3 + 3 pass Sandercock-type tandem Fabry-Perot interferometer (FPI) was used to measure the Brillouin spectra with a free spectral range (FSR) ±300 GHz over a wide temperature range from 298 K to 773 K. The exciting light source was a single frequency diode pump solid state (DPSS) laser with a wavelength of 532 nm and a power of about 100 mW. Each polarization of the scattered light, *x(yz*)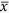 (VH) and *x(zz*)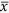 (VV), was measured separately by placing a circular polarizer in front of the entrance slit. The temperature of a sample was controlled by using a heating/cooling stage (THMS600, Linkam) with a stability of ± 0.1 °C. In backward scattering geometry, there is often so much elastic light that the photon counting system is saturated and so no signal is available for stabilizing the interferometer. In this case, it is necessary to reduce the laser intensity while scanning through the elastic peak. In order to do so, a double shutter system is integrated into the optical system directly behind the entrance pinhole to the spectrometer. By using these shutters, the elastic scattering is cut in the range between −60 to +60 GHz. Since the frequency of the acoustic modes exist between this ranges, therefore, the elastic and acoustic contributions were not observed when measuring the CP.

## Additional Information

**How to cite this article:** Helal, M. A. *et al*. Role of polar nanoregions with weak random fields in Pb-based perovskite ferroelectrics. *Sci. Rep.* **7**, 44448; doi: 10.1038/srep44448 (2017).

**Publisher's note:** Springer Nature remains neutral with regard to jurisdictional claims in published maps and institutional affiliations.

## References

[b1] Bellaiche L. & Vanderbilt D. Intrinsic Piezoelectric Response in Perovskite Alloys: PMN-PT versus PZT. Phys. Rev. Lett. 83, 1347–1350 (1999).

[b2] Fu H. & Cohen R. E. Polarization rotation mechanism for ultrahigh electromechanical response in single-crystal piezoelectrics. Nature 403, 281–283 (2000). 10659840 10.1038/35002022

[b3] Jin Y. M., Wang Y. U. & Khachaturyan A. G. Conformal miniaturization of domains with low domain-wall energy: monoclinic ferroelectric states near the morphotropic phase boundaries. Phys. Rev. Lett. 91, 197601 (2003). 14611617 10.1103/PhysRevLett.91.197601

[b4] Kutnjak Z., Petzelt J. & Blinc R. The giant electromechanical response in ferroelectric relaxors as a critical phenomenon. Nature 441, 956–959 (2006). 16791189 10.1038/nature04854

[b5] Xu G., Wen J., Stock C. & Gehring P. M. Phase instability induced by polar nanoregions in a relaxor ferroelectric system. Nat. Mater. 7, 562–566 (2008). 18469821 10.1038/nmat2196

[b6] Park S. E. & Shrout T. R. Ultrahigh strain and piezoelectric behavior in relaxor based ferroelectric single crystals. J. Appl. Phys. 82, 1804–1811 (1997).

[b7] Westphal V., Kleeman W. & Glinchuk M. D. Diffuse phase transitions and random-field induced domain states of the “relaxor” ferroelectric PbMg_1/3_Nb_2/3_O_3_. Phys. Rev. Lett. 68, 847–850 (1992). 10046008 10.1103/PhysRevLett.68.847

[b8] Stock C. . Universal static and dynamic properties of the structural transitions in PbZn_1/3_Nb_2/3_O_3_. Phys. Rev. B 69, 094104 (2004).

[b9] Grinberg I. & Rappe A. M. Local structure and macroscopic properties in PMN-PT and PZN-PT solid solutions. Phys. Rev. B 70, 220101 (2004).

[b10] Dkhil B. . Local and long range polar order in the relaxor-ferroelectric compounds PbMg_1/3_Nb_2/3_O_3_ and PbMg_0.3_Nb_0.6_Ti_0.1_O_3_. Phys. Rev. B 65, 024104 (2001).

[b11] Vakhrushev S., Zhukov S., Fetisov G. & Chernyshov V. The high-temperature structure of lead magnoniobate. J. Phys.: Condens. Matter 6, 4021–4027 (1994).

[b12] Ye Z.-G. . Development of ferroelectric order in relaxor (1−*x*) PbMg_1/3_Nb_2/3_O_3_-PbTiO_3_ (0 ≤ *x* ≤ 0.15). Phys. Rev. B 67, 104104 (2003).

[b13] Stock C. . Damped soft phonons and diffuse scattering in 40%Pb(Mg_1/3_Nb_2/3_)O_3_-60%PbTiO_3_. Phys. Rev. B 73, 064107 (2006).

[b14] Ko J.-H., Kojima S., Bokov A. A. & Ye Z. G. Light scattering study of acoustic phonon modes and central peaks in Pb [(Mg_1/3_Nb_2/3_)_0.45_Ti_0.55_]O_3_ single crystals. Appl. Phys. Lett. 91, 252909 (2007).

[b15] Ko J.-H., Kim T. H., Kojima S., Bokov A. A. & Ye Z.-G. Sound velocities and hypersonic dampings of Pb[(Mg_1/3_Nb_2/3_)_0.45_Ti_0.55_]O_3_ single crystals studied by Brillouin light scattering. J. Phys. Condens. Matter 22, 485902 (2010). 21406758 10.1088/0953-8984/22/48/485902

[b16] Ko J.-H. . Crossover in the mechanism of ferroelectric phase transition of Pb[(Mg_1/3_Nb_2/3_)_1−*x*_Ti_*x*_]O_3_ single crystals studied by Brillouin light scattering. Phys. Rev. B 82, 104110 (2010).

[b17] Helal M. A. & Kojima S. Relaxational and vibrational dynamics of 0.44Pb(Mg_1/3_Nb_2/3_)O_3_-0.56PbTiO_3_ single crystals probed by micro-Raman scattering. Ferroelectrics 487, 9–16 (2015).

[b18] Rahaman M. M., Imai T., Sakamoto T., Tsukada S. & Kojima S. Fano resonance of Li-doped KTa_1−*x*_Nb_*x*_O_3_ single crystals studied by Raman scattering. Sci. Rep. 6, 23898 (2016). 27049847 10.1038/srep23898 PMC4822152

[b19] Taniguchi H., Itoh M. & Fu D. Raman scattering study of the soft mode in Pb(Mg_1/3_Nb_2/3_)O_3_. J. Raman Spectrosc. 42, 706–714 (2011).

[b20] Fujii Y. . Raman tensor analysis of crystalline lead titanate by quantitative polarized spectroscopy. Ferroelectrics 462, 8–13 (2014).

[b21] Ge W., Zhu W. & Pezzotti G. Raman selection rules and tensor elements for PMN-0.3PT single crystal. Phys. Status Solidi B 246, 1340–1344 (2009).

[b22] Rafalovskyi I., Guennou M., Gregora I. & Hlinka J. Macroscopic lamellar heterophase pattern in Pb(Mg_1/3_Nb_2/3_)O_3_-PbTiO_3_ single crystals. Phys. Rev. B 93, 064110 (2016).

[b23] Shen M., Siu G. G., Xu Z. K. & Raman W. C. spectroscopy study of ferroelectric modes in [001]-oriented 0.67Pb(Mg_1∕3_Nb_2∕3_)O_3_-0.33PbTiO_3_ single crystals. Appl. Phys. Lett. 86, 252903 (2005).

[b24] Sivasubramanian V. & Kojima S. Brillouin scattering studies of acoustic phonon modes and central peak in single-crystal Pb(Sc_1/2_Ta_1/2_)O_3_. Phys. Rev. B 85, 054104 (2012).

[b25] Slodczyk A., Daniel P. & Kania A. Local phenomena of (1−*x*)PbMg_1/3_Nb_2/3_O_3_-*x*PbTiO_3_ single crystals (0 ≤ *x* ≤ 0.38) studied by Raman scattering. Phys. Rev. B 77, 184114 (2008).

[b26] Kania A., Daniel P. & Słodczyk A. Cubic–tetragonal–orthorhombic phase transition sequence in 0.5PbMg_1/3_Nb_2/3_O_3_-0.5PbTiO_3_ and 0.36PbMg_1/3_Nb_2/3_O_3_-0.64PbTiO_3_ single crystals. J. Phys. Condens. Matter 18, 9625–9641 (2006).

[b27] Toulouse J., Jiang F., Svitelskiy O., Chen W. & Ye Z.-G. Temperature evolution of the relaxor dynamics in Pb(Zn_1∕3_Nb_2∕3_)O_3_: A critical Raman analysis. Phys. Rev. B 72, 184106 (2005).

[b28] Jiang F. M. & Kojima S. Relaxation mode in 0.65Pb(Mg_1/3_Nb_2/3_)O_3_-0.35PbTiO_3_ relaxor single crystals studied by micro-Brillouin scattering. Phys. Rev. B 62, 8572–8575 (2000).

[b29] Nakata Y., Tsujimi Y., Katsuraya K., Iwata M. & Yagi T. Dynamical slowing down of polar nanoregion in relaxor-based ferroelctric 0.89Pb(Zn_2/3_Nb_1/3_)O_3_-0.11PbTiO_3_. Appl. Phys. Lett. 89, 022903 (2006).

[b30] Svitelskiy O. & Toulouse J. Translational and rotational mode coupling in disordered ferroelectrics KTa_1−*x*_Nb_*x*_O_3_ studied by Raman scattering. J. Phys. Chem. Solids 64, 665–676 (2003).

[b31] Ko J.-H., Kim D. & Kojima S. Central peaks, acoustic modes, and the dynamics of polar nanoregions in Pb[(Zn_1∕3_Nb_2∕3_)_*x*_Ti_1−*x*_]O_3_ single crystals studied by Brillouin spectroscopy. Phys. Rev. B 77, 104110 (2008).

[b32] Gorouya Y., Tsujimi Y., Iwata M. & Yagi T. Brillouin scattering study on relaxor ferroelectric Pb(Zn_1/3_Nb_2/3_)O_3_. Appl. Phys. Lett. 83, 1358 (2003).

[b33] Jeong I.-K. . Direct observation of the formation of polar nanoregions in Pb(Mg_1/3_Nb_2/3_)O_3_ using neutron pair distribution function analysis. Phys. Rev. Lett. 94, 147602 (2005). 15904113 10.1103/PhysRevLett.94.147602

[b34] Li F., Xu Z. & Zhang S. The effect of polar nanoregions on electromechanical properties of relaxor-PbTiO_3_ crystals: Extracting from electric-field-induced polarization and strain behaviors. Appl. Phys. Lett. 105, 122904 (2014).

[b35] La-Orauttapong D. . Neutron scattering study of the relaxor ferroelectric (1−*x*)Pb(Zn_1/3_Nb_2/3_)O_3_−*x*PbTiO_3_. Phys. Rev. B 67, 134110 (2003).

[b36] Katiyar R. S., Ryan J. F. & Scott J. F. Proton-phonon coupling in CsH_2_AsO_4_ and KH_2_AsO_4_. Phys. Rev. B 4, 2635–2638 (1971).

[b37] Long D. A. The Raman Effect: A unified Treatment of the Theory of Raman Scattering by MoleculesJohn Wiley & Sons, London (2001).

[b38] Hayes W. & Loudon R. Scattering of Light by CrystalsJohn Wiley & Sons, London (1978).

[b39] Lines M. & Glass A. Principles and Applications of Ferroelectrics and Related MaterialsOxford University Press, New York (2004).

[b40] Dmowski W. . Local lattice dynamics and the origin of the relaxor ferroelectric behavior. Phys. Rev. Lett. 100, 137602 (2008). 18517997 10.1103/PhysRevLett.100.137602

[b41] Strach T., Brunen J., Lederle B., Zegenhagen J. & Cardona M. Determination of the phase difference between the Raman tensor elements of the A_1g_-like phonons in SmBa_2_Cu_3_O_7−δ_. Phys. Rev. B 57, 1292–1297 (1998).

[b42] Svitelskiy O., Toulouse J., Yong G. & Ye Z.-G. Polarized Raman study of the phonon dynamics in Pb(Mg_1/3_Nb_2/3_)O_3_ crystal. Phys. Rev. B 68, 104107 (2003).

[b43] La-Orauttapong D., Svitelskiy O. & Toulouse J. Condensation and slow dynamics of polar nanoregions in lead relaxors. AIP Conf. Proc. 677, 98 (2003).

[b44] Ko J.-H., Kim D. H. & Kojima S. Correlation between the dynamics of polar nanoregions and temperature evolution of central peaks in Pb [(Zn_1/3_Nb_2/3_)_0.91_Ti_0.09_]O_3_ ferroelectric relaxors. Appl. Phys. Lett. 90, 112904 (2007).

[b45] Senn M. S., Keen D. A., Lucas T. C. A., Hriljac J. A. & Goodwin A. L. Emergence of long-range order in BaTiO_3_ from local symmetry-breaking distortions. Phys. Rev. Lett. 116, 207602 (2016). 27258883 10.1103/PhysRevLett.116.207602

[b46] Zalar B., Laguta V. V. & Blinc R. NMR evidence for the coexistence of order-disorder and displacive components in barium titanate. Phys. Rev. Lett. 90, 037601 (2003). 12570522 10.1103/PhysRevLett.90.037601

[b47] Vakhrushev S., Ivanov A. & Kulda J. Diffuse neutron scattering in relaxor ferroelectric PbMg_1/3_Nb_2/3_O_3_. Phys. Chem. Chem. Phys. 7, 2340–2345 (2005). 19785119 10.1039/b416454g

[b48] Vakhrushev S. B. & Shapiro S. M. Direct evidence of soft mode behavior near the Burns temperature in the PbMg_1/3_Nb_2/3_O_3_ relaxor ferroelectric. Phys. Rev. B 66, 214101 (2002).

[b49] Al-Zein A., Hlinka J., Rouquette J. & Hehlen B. Soft mode doublet in PbMg_1/3_Nb_2/3_O_3_ relaxor investigated with hyper-Raman scattering. Phys. Rev. Lett. 105, 017601 (2010). 20867477 10.1103/PhysRevLett.105.017601

[b50] Tsukada S. & Kojima S. Broadband light scattering of two relaxation processes in relaxor ferroelectric 0.93Pb(Zn_1/3_Nb_2/3_)O_3_-0.07PbTiO_3_ single crystals. Phys. Rev. B 78, 144106 (2008).

[b51] Landau L. D. & Lifshitz E. M. Theory of ElasticityButterworth-Heinemann, Oxford, UK (1986).

[b52] Tsukada S. . Broadband inelastic light scattering of a relaxor ferroelectric 0.71Pb(Ni_1∕3_Nb_2∕3_)O_3_-0.29PbTiO_3_. Appl. Phys. Lett. 89, 212903 (2006).

[b53] Kuok M. H., Ng S. C., Fan H. J., Iwata M. & Ishibashi Y. Hypersonic frequency softening and relaxation in relaxor ferroelectric 0.8Pb(Zn_1/3_Nb_2/3_)O_3_-0.2PbTiO_3_. Solid State Commun. 118, 169–172 (2001).

[b54] Luo H., Xu G., Xu H., Wang P. & Yin Z. Compositional homogeneity and electrical properties of lead magnesium niobate titanate single crystals grown by a modified bridgman technique. Jpn. J. Appl. Phys. 39, 5581–5585 (2000).

[b55] Kojima S. Gigahertz acoustic spectroscopy by micro-Brillouin scattering. Jpn. J. Appl. Phys. 49, 07HA01 (2010).

